# Current Status of Immune Checkpoint Inhibitor Immunotherapy for Lung Cancer

**DOI:** 10.3389/fonc.2021.704336

**Published:** 2021-08-18

**Authors:** Wei Xiong, Yunfeng Zhao, He Du, Xuejun Guo

**Affiliations:** ^1^Department of Pulmonary and Critical Care Medicine, Xinhua Hospital, Shanghai Jiaotong University School of Medicine, Shanghai, China; ^2^Department of Pulmonary and Critical Care Medicine, Punan Hospital, Shanghai, China; ^3^Department of Medical Oncology, Shanghai Pulmonary Hospital, Tongji University School of Medicine, Shanghai, China

**Keywords:** lung cancer, immunotherapy, immune checkpoint inhibitors, programmed death-ligand 1, current status

## Abstract

Immunotherapy is a major breakthrough in the treatment of cancer in recent years. Immune checkpoint inhibitors (ICIs) including programmed death-ligand 1 (*PD-L1*)/programmed death-1 (*PD-1*) and cytotoxic T-lymphocyte antigen-4 (*CTLA-4*) have been used for different histologic types of cancer including primary lung cancer that represents the most common and fatal cancer globally. Among ICI immunotherapy agents, atezolizumab, durvalumab, ipilimumab, nivolumab, and pembrolizumab are currently used as standard-of-care (SOC) treatment for metastatic or earlier stages of lung cancer. Major issues of ICI immunotherapy in lung cancer comprise the use of immune biomarkers prior to ICI therapy, selection of ICI agents, combination of ICIs/chemotherapy, combination of ICIs/radiotherapy, sequence of tyrosine kinase inhibitor (TKI) targeted therapy and ICI immunotherapy, sequence of chemotherapy and ICI immunotherapy, treatment duration of ICI regimen and ICI therapy for different histopathology, stage, *PD-L1*, and performance status. Based on the contemporary major clinical trials and authoritative guidelines, the objective of this review is to present an overview of the current status of ICI immunotherapy in lung cancer.

## Introduction

Tumor cells escape the immune system when they develop resistance to antitumor immune response through multiple mechanisms including alteration of antigens, manipulation of cytokine, and upregulation of immune checkpoint proteins (1). Immunotherapies including immune checkpoint inhibitors (ICIs), adoptive cell therapy, oncolytic viruses, and cancer vaccines reactivate antitumor immune response and overcome the pathways leading to tumor escape (2). Among the abovementioned immunotherapeutic agents, ICIs especially programmed death-ligand 1 (*PD-L1*)/programmed death-1 (*PD-1*) have been frequently proven to be effective and safe in the immunotherapy of cancer including primary lung cancer, which is a well-known common and fatal cancer globally.

**Figure 1:**
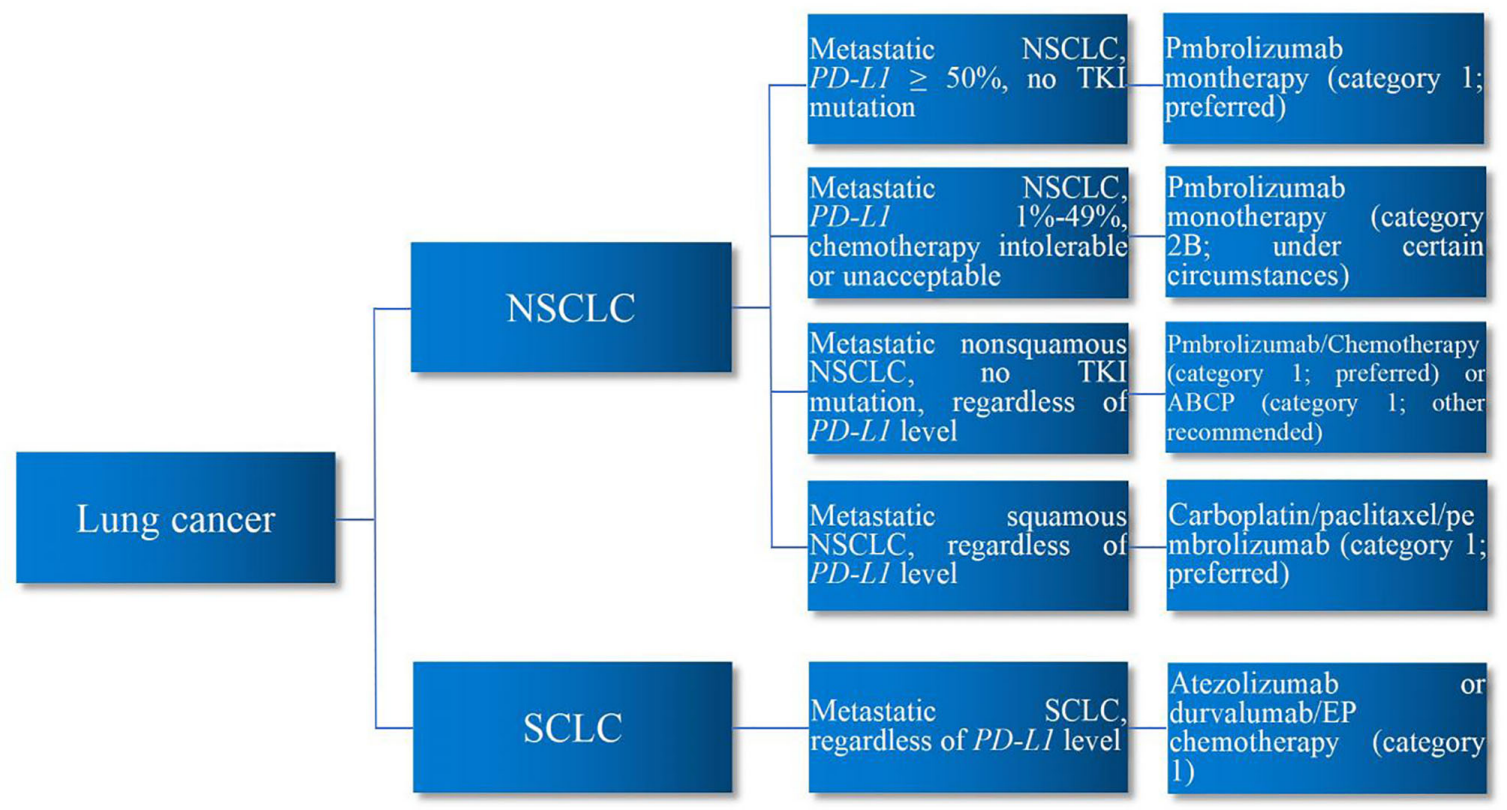
First-Line ICI Immunotherapy for Lung Cancer. In the first-line ICI immunotherapy of lung cancer, for metastatic NSCLC patients with *PD-L1* ≥50% without *TKI* mutation, pembrolizumab monotherapy is preferred. For metastatic NSCLC patients with *PD-L1* 1%–49% who are intolerable or unacceptable to chemotherapy, pembrolizumab monotherapy may be considered. Regardless of *PD-L1* level, for metastatic non-squamous NSCLC patients without *TKI* mutation, pembrolizumab/chemotherapy is preferred or ABCP regimen may be considered. Regardless of *PD-L1* level, for metastatic squamous NSCLC patients, carboplatin/paclitaxel/pembrolizumab regimen is preferred. Regardless of *PD-L1* level, for metastatic SCLC patients, atezolizumab or durvalumab/EP chemotherapy is preferred. ICIs, immune checkpoint inhibitors; NSCLC, non-small cell lung cancer; SCLC, small cell lung cancer; *PD-L1*, programmed death-ligand 1; *TKI*, tyrosine kinase inhibitor; ABCP, atezolizumab/bevacizumab/carboplatin/paclitaxel; EP, etoposide/platinum.

*PD-L1* system prevents overactivation of T cells to maintain homeostasis and avoid autoimmunity (3). Developing tumor cells take advantage of this to upregulate the *PD-L1* pathway, reduce T-cell function and proliferation, and promote the development of T-cell exhaustion, thereby escaping immune destruction. *PD-L1*-related ICIs deactivate such signaling pathway to reinvigorate T-cell immune response to tumor cells, thereby eliminating them. Nevertheless, autoreactive T cells can also be unleashed by ICIs, leading to immune-related adverse events (irAEs) in such process (4). Accordingly, whether, when, and how to use ICI immunotherapy are the essential questions in the immunotherapy of patients with lung cancer. Major issues of ICI immunotherapy in lung cancer comprise the use of immune biomarker prior to ICI therapy, selection of ICI agents, combination of ICIs/chemotherapy, combination of ICIs/radiotherapy, sequence of tyrosine kinase inhibitor (TKI) targeted therapy and ICI immunotherapy, sequence of chemotherapy and ICI immunotherapy, treatment duration of ICI regimen, and ICI therapy for different histopathology, stage, *PD-L1*, and performance status. Encompassing the aforementioned issues, this review aims to present an overview of the current status of ICI immunotherapy in lung cancer based on the current major clinical trials and authoritative guidelines. Major clinical trials demonstrating the efficacy of ICI immunotherapy for lung cancer are summarized in **Table 1**. First-line ICI immunotherapy for lung cancer are demonstrated in **Figure 1**.

**Table 1:** Major Clinical Trials Demonstrating the Efficacy of ICI Immunotherapy for Lung Cancer.

Type of lung cancer	Title of trial	Characteristics of patients	Agents
Metastatic NSCLC	KEYNOTE-024	*PD-L1* ≥50%	Pembrolizumab *vs.* platinum-based chemotherapy
First-line	KEYNOTE-189	Non-squamous	Pembrolizumab *vs.* placebo+platinum and pemetrexed
Second-line and above	KEYNOTE-407	Squamous	Pembrolizumab *vs.* placebo+carboplatin and paclitaxel or nab-paclitaxel
	KEYNOTE-042	*PD-L1* ≥1%	Pembrolizumab *vs.* platinum-based chemotherapy
	CheckMate 227	All *PD-L1* level	Nivolumab+ipilimumab *vs.* nivolumab+chemotherapy *vs.* chemotherapy
	CheckMate 9LA	Advanced NSCLC	Nivolumab+opilimumab+chemotherapy *vs.* chemotherapy
	IMpower150	Non-squamous without EGFR or ALK alteration	Atezolizumab+carboplatin+paclitaxel (ACP) *vs.* bevacizumab+carboplatin+paclitaxel (BCP) *vs.* atezolizumab+BCP (ABCP)
	IMpower130	Non-squamous without EGFR or ALK alteration	Atezolizumab+carboplatin+nab-paclitaxel *vs.* chemotherapy
	IMpower110	*PD-L1 ≥1%* without EGFR or ALK alteration	Atezolizumab *vs.* platinum-based chemotherapy
	CheckMate 017	Squamous	Nivolumab *vs.* docetaxel
	CheckMate 057	Non-squamous	Nivolumab *vs.* docetaxel
	POPLAR	Previously treated	Atezolizumab *vs.* docetaxel
	OAK	advanced NSCLC	Atezolizumab *vs.* docetaxel
		Previously treated	
		advanced NSCLC	
Locally advanced NSCLC	PACIFIC	Locally advanced unresectable NSCLC	Durvalumab *vs.* placebo
Extensive-stage SCLC	KEYNOTE-604	First-line	Atezolizumab *vs.* placebo + platinum and etoposide
	CASPIAN	First-line	Durvalumab+platinum and etoposide *vs.* platinum and etoposide
	KEYNOTE-604	First-line	Pembrolizumab *vs.* placebo+platinum and etoposide
Recurrent SCLC	CheckMate 032	Previously treated SCLC	Nivolumab *vs.* nivolumab plus ipilimumab
	KEYNOTE-028	Previously treated SCLC with *PD-L1* expression	Pembrolizumab

ICIs, immune checkpoint inhibitors; NSCLC, non-small cell lung cancer; PD-L1, programmed death-ligand 1; EGFR, epidermal growth factor receptor; ALK, anaplastic lymphoma kinase; SCLC, small cell lung cancer.

## Non-Small Cell Lung Cancer

### Advanced or Metastatic Non-Small Cell Lung Cancer

ICI immunotherapy has been extensively used in the standard clinical practice of patients with NSCLC especially at the advanced and metastatic stage, despite the challenges of irAEs (5, 6). In the earliest studies in which most regimens are now considered to be second-line and above, patients with advanced non-squamous (CheckMate 057) or squamous cell (CheckMate 017) NSCLC previously treated with chemotherapy had better overall survival (OS) or progression-free survival (PFS) with nivolumab than with docetaxel, regardless of *PD-L1* expression level (7, 8). In the KEYNOTE-010 trial, pembrolizumab increased OS in previously treated advanced NSCLC patients with *PD-L1*expression on at least 50% of tumor cells (9). In the OAK trial, atezolizumab resulted in a better clinically relevant OS than docetaxel in previously treated NSCLC, regardless of *PD-L1* expression or histology (10).

Along with the rise of the ICI immunotherapy, *PD-L1* expression or tumor proportion score (TPS) that serves as a predictive biomarker to identify patients who will likely benefit from immunotherapeutic agents has been recognized. In the KEYNOTE-001 trial, *PD-L1* expression in at least 50% of tumor cells suggested improved efficacy of pembrolizumab in patients with advanced NSCLC (11). The POPLAR trial also confirmed that *PD-L1* expression was predictive for the benefit of atezolizumab in patients with previously treated NSCLC (12). For patients with previously untreated advanced NSCLC, the KEYNOTE-024 study reported that pembrolizumab resulted in significantly longer PFS and OS as well as fewer adverse events than platinum-based chemotherapy in those who had *PD-L1* expression on at least 50% of tumor cells, establishing the role of *PD-L1* TPS in the initial treatment of NSCLC (13). Although blood-based tumor mutational burden (TMB), which is an approximate measure of the total number of somatic mutations, may correlate with high neoantigen levels that can activate an antitumor immune response, it is not an ideal immune biomarker that can accurately identify the proper patients who will respond to ICI immunotherapy (14). Besides, the lack of standard cutoff value and standardization of TMB measurements across laboratories is another concern for its application in the ICI immunotherapy of lung cancer (15). Nevertheless, the technical feasibility of assessing TMB by adopting cell blocks (CBs) has been proven recently (16). In addition, in a systematic review and meta-analysis, high-TMB patients receiving immune-oncology (IO) agents were associated with improved overall response rate (ORR) [relative risk (RR) 1.37, 95% CI 1.13–1.66], PFS [hazard ratio (HR) 0.69, 95% CI 0.61–0.79], and OS (HR 0.67, 95% CI 0.59–0.77), whereas for low-TMB patients, the IO strategy did not lead to any significant benefit in survival and activity (17).

Besides *PD-L1* TPS and TMB, in a study assessing the prognostic and predictive roles of neutrophil–lymphocyte ratio (NLR), lactate dehydrogenase (LDH), and advanced lung cancer inflammation index (ALI) in 120 patients with small-cell lung cancer (SCLC) (n = 110) and large cell neuroendocrine carcinoma (NEC) (n = 10), OS was better in patients with pretreatment NLR <1.93 (p = 0.0002), LDH <600 U/L (p = 0.03), and ALI ≥34 (p = 0.0065). At a multivariate analysis, LDH levels were independently associated with OS. Patients with higher NLR (>1.93) had an increased probability of tumor progression (p = 0.045). The systemic inflammatory biomarkers may be used to understand survival differences among lung cancer patients in the ICI setting (18).

Among the approved ICI agents, pembrolizumab-based regimen is highly favored in the ICI immunotherapy of NSCLC. In the prolonged follow-up of the KEYNOTE-024 study, first-line pembrolizumab monotherapy continued to outperform chemotherapy on OS in patients with previously untreated advanced NSCLC without epidermal growth factor receptor (*EGFR*) or anaplastic lymphoma kinase (*ALK*) aberrations (19). Then, the KEYNOTE-042 study suggested that pembrolizumab monotherapy was an efficacious first-line therapy to patients with locally advanced or metastatic NSCLC without *EGFR* or *ALK* alterations or high *PD-L1* TPS who may not tolerate chemotherapy (20). The analysis of KEYNOTE-001 study also indicated that pembrolizumab monotherapy that possessed durable anticancer activity resulted in high 5-year OS rates along with long-term safety among NSCLC patients with or without previous treatment. The 5-year OS exceeded 25% in patients with previous treatment and 29.6% in treatment-naive patients (21).

With respect to ICI/chemotherapy regimens, in the PROLUNG Phase 2 trial, ORR was higher in patients receiving pembrolizumab plus docetaxel than that in patients receiving docetaxel (42.5% *vs.* 15.8%, OR 3.94, 95% CI 1.34–11.54, p = 0.01). PFS was longer in patients receiving pembrolizumab plus docetaxel (9.5 months, 95% CI 4.2–not reached) than that in those receiving docetaxel (3.9 months, 95% CI 3.2–5.7) (HR 0.24, 95% CI 0.13–0.46, p < 0.001). No severe safety issues were encountered. The combination of pembrolizumab and docetaxel significantly improved ORR and PFS in patients with advanced NSCLC and previous progression after platinum-based chemotherapy, with or without EGFR variations (22).

With regard to ICI/radiotherapy regimens, in a pooled analysis of two randomized trials, best abscopal response rate (ARR) was 19.7% with pembrolizumab *vs.* 41.7% with pembrolizumab plus radiotherapy (OR 2.96, 95% CI 1.42–6.20, p = 0.0039), whereas best abscopal disease control rate (ACR) was 43.4% with pembrolizumab *vs.* 65.3% with pembrolizumab plus radiotherapy (OR 2.51, 95% CI 1.28–4.91, p = 0.0071). Median PFS was 4.4 (2.9–5.9) months with pembrolizumab alone *vs.* 9.0 months (6.8–11.2) with pembrolizumab plus radiotherapy (HR 0.67, 95% CI 0.45–0.99, p = 0.045), whereas median OS was 8.7 (6.4–11.0) months with pembrolizumab *vs.* 19.2 (14.6–23.8) months with pembrolizumab plus radiotherapy (HR 0.67, 95% CI 0.54–0.84; p=0.0004). No new safety concerns were found in the pooled analysis (23).

For NSCLC patients with different PS, in a phase 2 trial (NCT02085070), pembrolizumab had therapeutic efficacy in advanced NSCLC patients with PS <2 (24). For NSCLC patients with PS ≥2 who received pembrolizumab in the PePS2 trial, the incidence for durable clinical benefit (DCB) was 38% (95% CI 21%–57%) in first-line patients (n = 24) and 36% (22%–52%) in subsequent-line patients (n = 36). DCB was 22% (11%–41%) in patients with a TPS less than 1% (n = 27), 47% (25%–70%) in patients with a TPS of 1%–49% (n = 15), and 53% (30–75) in patients with a TPS of 50% or greater (n = 15). Toxicity was observed in 28% (19%–41%) of patients (25). When PS score is 2 or more, the efficacy of ICI immunothreapy seems to be positively correlated with the TPS of *PD-L1*.

For NSCLC patients with all *PD-L1* categories, the KEYNOTE-189 study demonstrated for the first time that the addition of pembrolizumab to standard chemotherapy resulted in significantly longer OS and PFS than chemotherapy alone in patients with metastatic non-squamous NSCLC without previous treatment or *EGFR* or *ALK* mutations, regardless of *PD-L1* expression (26). In the Impower150 study that was also regardless of *PD-L1* expression, the combination of atezolizumab, bevacizumab, plus chemotherapy significantly improved PFS and OS among patients with metastatic non-squamous NSCLC, regardless of *EGFR* or *ALK* genetic mutations (27).

Besides pembrolizumab, atezolizumab is also frequently used in the ICI therapy for patients with NSCLC. In the IMpower130 trial, as first-line treatment, atezolizumab plus chemotherapy resulted in an improvement in OS and PFS without new safety concerns compared with chemotherapy alone for patients with stage IV non-squamous NSCLC without *ALK* or *EGFR* mutations (28). In the IMpower110 trial, for patients without *EGFR* or *ALK* mutations who had the highest expression of *PD-L1*, the median OS was 20.2 months in the atezolizumab group and 13.1 months in the chemotherapy group (HR for death, 0.59; p = 0.01). Adverse events presented in 90.2% of the patients with atezolizumab and in 94.7% of those with chemotherapy (29).

With progressively increasing frontline regimens for patients with NSCLC, optimal sequencing of therapy becomes an open question in this field. Of note, the sequence of administration of ICI immunotherapy and TKI targeted therapy is one of the concerns. For NSCLC patients with targetable driver mutations, a retrospective study showed that *PD-L1* inhibitor followed by osimertinib instead of other *EGFR*–TKIs resulted in severe irAEs, especially among patients who received *PD-L1* blockade recently, suggesting the importance of appropriate sequence of ICI immunotherapy and osimertinib (30). In addition, CAURAL brief report was terminated prematurely due to an increased incidence of interstitial lung disease in osimertinib plus durvalumab combination therapy group compared with that in osimertinib monotherapy group (31). Moreover, along with an increased risk of toxicity, a phase 2 study demonstrated that pembrolizumab lacked efficacy in *EGFR*-mutant, *PD-L1*-positive, and TKI-naive patients with advanced NSCLC, including those with *PD-L1* ≥50% (32). Based on the abovementioned results, in the setting of NSCLC patients with both *EGFR* mutation and *PD-L1* positivity, immunotherapy is suggested to be withheld temporarily to ensure both efficacy and safety, whereas it still remains an alternative once patients develop TKI resistance.

The decision of sequence and timing of chemotherapy/immunotherapy is also a challenge. In the KEYNOTE-189, KEYNOTE-407, and IMpower150 trials, combination of chemotherapy plus immunotherapy in patients with high *PD-L1* expression led to 60% or greater ORR and less progression. Nevertheless, its effect on long-term survival remains unknown. Although toxicity with grade 3 through 5 adverse events increased in the range of 58% to 68% along with the addition of chemotherapy to immunotherapy, the overall safety and tolerability of chemoimmunotherapy are comparable to those of chemotherapy alone (24, 25, 33). Therefore, for patients with true requirements and tolerance for combined therapy, immediate chemoimmunotherapy is recommended for optimal disease control and improved efficacy; otherwise, sequential treatment of single-agent immunotherapy followed by chemotherapy at progression is recommended. Nevertheless, the optimal sequence and timing of chemotherapy/immunotherapy have been understudied to date.

For NSCLC patients with low *PD-L1* expression level who are not willing to receive chemotherapy, the CheckMate 227 trial demonstrated that first-line treatment with nivolumab plus ipilimumab that was a monoclonal antibody inhibiting cytotoxic T-lymphocyte antigen-4 (*CTLA-4*) resulted in a longer duration of overall survival than chemotherapy alone without long-term safety concerns, regardless of *PD-L1* expression level. The second co-primary endpoint demonstrated that the median OS was longer in the immunotherapy group than that in the chemotherapy group across all *PD-L1* expression levels (34). The CheckMate 227 also showed that PFS was significantly longer with first-line nivolumab plus ipilimumab than with chemotherapy in patients with NSCLC, regardless of *PD-L1* expression level. The results validated the role of nivolumab plus ipilimumab in NSCLC (35). Recently, in the CheckMate 9LA trial, OS was significantly longer in patients receiving nivolumab plus ipilimumab with two cycles of chemotherapy than that in those receiving four cycles of chemotherapy alone [14.1 (13.2–16.2) *vs.* 10.7 (9.5–12.4), HR 0.69 (0.55–0.87), p = 0.00065]. Median OS was 15.6 (13.9–20.0) months in the experimental group *vs.* 10.9 (9.5–12.6) months in the control group [HR 0.66 (0.55–0.80)]. Serious irAEs occurred in 106 (30%) patients in the experimental group and 62 (18%) in the control group (36).

Similar to non-squamous NSCLC, for patients with advanced squamous NSCLC, immunotherapy also has an excellent efficacy. Besides CheckMate 017 (8), in the KEYNOTE-407 trial, for patients with untreated metastatic squamous NSCLC, the median OS was 15.9 months (95% CI 13.2–not reached) in the pembrolizumab–combination group and 11.3 months (95% CI 9.5–14.8) in the placebo–combination group (HR for death 0.64, 95% CI 0.49–0.85, p < 0.001). The OS benefit was consistent regardless of the level of *PD-L1* expression. The median PFS was 6.4 months (95% CI 6.2–8.3) in the pembrolizumab–combination group and 4.8 months (95% CI 4.3–5.7) in the placebo–combination group (HR for disease progression or death 0.56, 95% CI 0.45–0.70, p < 0.001). Adverse events of grade 3 or higher were similar between the pembrolizumab–combination group and the placebo–combination group (33).

With respect to the treatment duration of ICI immunotherapy, the CheckMate 153 trial demonstrated that the median PFS of patients with previously treated advanced NSCLC was longer with continuous nivolumab (24.7 months) than with 1-year fixed duration of nivolumab (9.4 months) (HR 0.56, 95% CI 0.37–0.84) without new safety signals. It suggested that continuing nivolumab regimen beyond 1 year improved outcomes in contrast with the fixed 1-year immunotherapy (37).

With respect to the combination of *PD-1/CTLA-4* inhibitors, a meta-analysis demonstrated that the IO+chemotherapy (CT) regimen resulted in a significant increase of ORR, among single-agent IO, IO+CT, and combination of *PD-1/CTLA-4* inhibitors (ComboIO), for the first-line treatment of patients with advanced NSCLC. For patients with negative *PD-L1* expression, no significant differences were found between the IO+CT and the dual ICIs in terms of activity and efficacy. However, the dual ICIs were better tolerated than IO+CT. Accordingly, the role of the combination of *PD-1/CTLA-4* inhibitors is currently limited in patients with *PD-L1*-high and/or *PD-L1*-low advanced NSCLC, whereas it may be considered a potentially effective and tolerable option in *PD-L1*-negative subgroups (38).

### Locally Advanced or Early-Stage Non-Small Cell Lung Cancer

For locally advanced NSCLC, the PACIFIC trial demonstrated a median PFS improvement from 5.6 months with placebo (95% CI 4.6–7.8 months) to 16.8 months with durvalumab (95% CI 13.0–18.1 months) in patients who had completed definitive chemoradiotherapy (CRT) for locally advanced NSCLC. *PD-L1* expression is not required to initiate durvalumab after CRT (39). The PACIFIC trial also showed that durvalumab therapy resulted in significantly longer OS than that in placebo (stratified HR for death 0.68; 99.73% CI 0.47–0.997, p = 0.0025). In addition, this regimen was well tolerated, although serious pneumonitis attributable to immunotherapy was encountered (40). In the PACIFIC trial, the updated OS remained consistent with that of a previous study (HR 0.69, 95% CI 0.55–0.86). The rates of 12-, 24-, and 36-month OS between the durvalumab group and the placebo group were 83.1% *vs.* 74.6%, 66.3% *vs.* 55.3%, and 57.0% *vs.* 43.5%, respectively. The PACIFIC regimen has been established as an SOC in patients with unresectable stage III NSCLC (41). Based on the results of the PACIFIC trial, ongoing studies are now exploring how to manipulate the immune system so as to improve outcomes by preventing recurrence for earlier stages of NSCLC.

For resectable NSCLC, a clinical trial (NCT02259621) showed that neoadjuvant nivolumab resulted in a major pathological response in 45% of resected tumors without side effects and delay of surgery (42). In that pilot study, a 4-week immunotherapy treatment period before surgery was safe and well tolerated, with 20 of 21 patients undergoing subsequent complete resection. Initial efficacy of neoadjuvant immunotherapy in resectable disease was quite promising based on 45% of major pathological response (MPR), which was defined as <10% viable tumor in the resected surgical specimen (42, 43). Recently, in the NADIM trial, the addition of neoadjuvant nivolumab to platinum-based chemotherapy achieved a 77.1% (95% CI 59.9–87.7) PFS at 24 months in patients with resectable stage IIIA NSCLC, without irAEs resulting in surgery delays or deaths (44). In another single-arm phase 2 trial, atezolizumab plus carboplatin and nab-paclitaxel achieved a major pathological response and manageable irAEs that did not compromise surgical resection, being a potential neoadjuvant regimen for resectable NSCLC (45).

## Small Cell Lung Cancer

### Extensive-Stage Small Cell Lung Cancer

The addition of immunotherapy to the traditional treatment options for SCLC has brought about great improvement for the management of this aggressive disease in decades. *PD-1*-targeted immunotherapy combined with chemotherapy has been defined to be the frontline SOC for extensive-stage SCLC nowadays.

In the IMpower133 trial, at a median follow-up of 13.9 months, the median OS was 12.3 months among 201 patients in the atezolizumab group and 10.3 months among 202 patients in the placebo group (HR for death 0.70, 95% CI 0.54–0.91, p = 0.007). The median PFS was 5.2 months and 4.3 months, respectively (HR for disease progression or death 0.77, 95% CI 0.62–0.96, p = 0.02). The safety of atezolizumab plus carboplatin and etoposide was consistent with that in the previous studies (46). In the CASPIAN study, durvalumab plus platinum–etoposide was associated with a significant improvement in OS, with an HR of 0.73 (95% CI 0.59–0.91, p = 0.0047). Median OS was 13.0 months (95% CI 11.5–14.8) in 268 patients receiving durvalumab plus platinum–etoposide *vs.* 10.3 months (95% CI 9.3–11.2) in 269 patients receiving platinum–etoposide. The patients who were alive at 18 months was 34% (26.9%–41.0%) in durvalumab group *vs.* 25% (18.4%–31.6%) in the non-durvalumab group. All-cause adverse events of grade 3 or 4 and the mortality resulting from adverse events were similar between the durvalumab plus platinum–etoposide group and the platinum–etoposide group (47).

Besides atezolizumab and durvalumab, the KEYNOTE-604 study demonstrated that pembrolizumab plus etoposide and platinum (EP) significantly improved PFS (HR 0.75, 95% CI 0.61–0.91, p = 0.0023). Twelve-month PFS estimates were 13.6% with pembrolizumab plus EP and 3.1% with placebo plus EP, whereas 24-month OS estimates were 22.5% and 11.2%, respectively. ORR was 70.6% with pembrolizumab plus EP and 61.8% with placebo plus EP. No unexpected toxicities were encountered with pembrolizumab plus EP (48). Nevertheless, for other agents, the CheckMate 451 trial revealed that OS was not prolonged by maintenance therapy with nivolumab plus ipilimumab for patients with extensive SCLC who did not progress after first-line chemotherapy (49).

Recently, a randomized phase II clinical trial demonstrated that nivolumab+CE (platinum–etoposide) regimen significantly improved PFS compared to CE with HR 0.65 (95% CI 0.46–0.91, p = 0.012) for the first-line treatment of patients with extensive-stage SCLC. The OS also improved with nivolumab+CE *vs.* CE with HR 0.67 (95% CI 0.46–0.98, p = 0.038). The ORR was 52.29% *vs.* 47.71% between nivolumab+CE and CE groups. The addition of nivolumab to CE as first-line treatment significantly improved PFS and OS without new safety concerns for patients with extensive-stage SCLC (50).

### Limited-Stage Small Cell Lung Cancer

Approximately one-fourth of patients with SCLC are diagnosed at limited stage, for which SOC therapy is CRT followed by prophylactic cranial radiation (PCI). Nevertheless, the 5-year OS rate still remains at 20%–25% with this regimen (51). The evidence with respect to the ICI immunotherapy in limited-stage SCLC is scarce. Nevertheless, several clinical trials such as ACHILES, STIMULI, ADRIATIC, NRG-LU005, and CLOVER are in progress for patients with SCLC at limited stage.

### Recurrent Small Cell Lung Cancer

For recurrent SCLC, the role of immunotherapy is evolving. In the CheckMate 032 trial, objective response was achieved in 10 of 98 patients with recurrent SCLC (10%) receiving nivolumab at a dose of 3 mg/kg every 2 weeks, 14 of 61 patients (23%) receiving nivolumab at a dose of 1 mg/kg plus ipilimumab at a dose of 3 mg/kg, and 10 of 54 patients (19%) receiving nivolumab at a dose of 3 mg/kg plus ipilimumab at a dose of 1 mg/kg. Both nivolumab monotherapy and nivolumab plus ipilimumab showed anticancer efficacy with durable responses and reliable safety in patients with previously treated SCLC (52). In the CheckMate 032 trial, ORR was 21.9% in 96 patients who received nivolumab plus ipilimumab and 11.6% in 147 patients who received nivolumab (OR 2.12, 95% CI 1.06–4.26, p = 0.03). For long-term OS, median OS was 5.7 (3.8–7.6) *vs.* 4.7 months (3.1–8.3). Twenty-four-month OS rates were 17.9% with nivolumab and 16.9% with nivolumab plus ipilimumab. Grade 3 to 4 treatment-related adverse event rates were 12.9% with nivolumab and 37.5% with nivolumab plus ipilimumab (53). Based on the results of the KEYNOTE-028 trial, single-agent pembrolizumab can be regarded as an approved third-line regimen for SCLC (54).

## Guidelines

### Non-Small Cell Lung Cancer

With respect to ICI immunotherapy, in the latest National Comprehensive Cancer Network (NCCN) guideline insights for NSCLC, the appropriate use of *PD-1/PD-L1* inhibitors is determined by using levels of *PD-L1* expression. Although TMB is a promising biomarker for the efficacy of ICI immunotherapy, the panel currently does not recommend the measurement of TMB levels before the decision on the use of ICIs such as nivolumab/ipilimumab or pembrolizumab for NSCLC. Optimal management of the unique toxicities associated with immunotherapy is also critical for satisfactory prognoses. Pembrolizumab monotherapy is strongly recommended as a first-line regimen for patients with metastatic non-squamous NSCLC, squamous cell NSCLC, *PD-L1* expression levels of ≥50%, no contraindications to immunotherapy, and non-squamous NSCLC with negative results of *EGFR*, *ALK*, *ROS1*, or *BRAF* genetic alterations by the panel. Pembrolizumab monotherapy is moderately recommended for patients with metastatic NSCLC and *PD-L1* levels of 1%–49% who cannot tolerate or accept platinum-based chemotherapy. Pembrolizumab/chemotherapy regimens are strongly recommended as first-line regimens for patients with metastatic non-squamous NSCLC, no contraindications to immunotherapy, and negative results of *EGFR*, *ALK*, *ROS1*, or *BRAF* genetic alterations, regardless of *PD-L1* expression levels. Of which, pembrolizumab/carboplatin/pemetrexed is strongly recommended. The panel also moderately recommends the ABCP (atezolizumab/bevacizumab/carboplatin/paclitaxel) tetragenous regimen as first-line option for patients with metastatic non-squamous NSCLC, no contraindications to immunotherapy or bevacizumab, and negative results of *EGFR*, *ALK*, *ROS1*, or *BRAF* genetic alterations, regardless of *PD-L1* expression levels. For patients with metastatic squamous cell NSCLC without contraindications to immunotherapy, carboplatin/paclitaxel/pembrolizumab regimens are strongly recommended as first-line option, regardless of *PD-L1* expression levels (5, 55).

With respect to the latest American Society of Clinical Oncology (ASCO) and Ontario Health (Cancer Care Ontario) joint guidelines for NSCLC, recommendations of ICI immunotherapy apply to patients who lack driver alterations in *EGFR* or *ALK*, as per the condition of histology, *PD-L1* status, and/or the presence or absence of contraindications. For patients with *PD-L1* ≥50% and non-squamous NSCLC, the panel recommends pembrolizumab monotherapy, pembrolizumab/carboplatin/pemetrexed, or ABCP regimens. For non-squamous NSCLC patients with either negative (0%) or low positive (1%–49%) *PD-L1*, the panel recommends pembrolizumab/carboplatin/pemetrexed, atezolizumab/carboplatin/nab-paclitaxel, ABCP regimens, or platinum-based or non-platinum-based two-drug combination chemotherapy. For patients with high *PD-L1* expression ≥50% and squamous cell NSCLC, the panel recommends pembrolizumab monotherapy or pembrolizumab/carboplatin/paclitaxel regimens. For squamous cell NSCLC patients with either negative (0%) or low positive *PD-L1* (1%–49%), the panel recommends pembrolizumab/carboplatin/paclitaxel or chemotherapy. For both non-squamous and squamous NSCLC, pembrolizumab monotherapy is also an option for select patients with low positive *PD-L1* (56).

### Small Cell Lung Cancer

With respect to ICI therapy, in the latest NCCN guideline insights for SCLC, the panel recommends nivolumab (category 2A) and nivolumab+ipilimumab (category 2A) as subsequent options for SCLC patients who have experienced relapse within 6 months after primary therapy. Pembrolizumab is not highly recommended for SCLC. Of note, unique irAEs other than traditional chemotherapy are associated with ICIs. If that happens, ICIs should be withheld or discontinued, then high-dose corticosteroids are generally recommended based on the severity of irAEs (57), for which the odds requiring hospitalization were higher with younger age, and the treatment of combined ICI therapies. Such factors may guide the treatment and management decisions with respect to irAEs (58). In the latest NCCN guidelines of SCLC, carboplatin/etoposide/atezolizumab or durvalumab (category 1) was the first choice of first-line systemic therapy for extensive-stage SCLC, followed by ICI maintenance therapy.

## Conclusions

ICI therapy has been widely used in patients with lung cancer. ICI treatment options at disease progression with or without traditional chemotherapy options have become an SOC for lung cancer. Nevertheless, how to determine whether a patient can benefit from ICI therapy is crucial. Although TMB is also a promising immune biomarker for the efficacy estimate of ICI immunotherapy, the appropriate use of *PD-1/PD-L1* ICIs in lung cancer is primarily determined by using levels of *PD-L1* TPS to date. For NSCLC, TKI driver gene testing is recommended prior to the decision of ICI immunotherapy. Priority is given to TKI targeted therapy under the circumstances that patients have TKI driver gene mutation with or without a high level of *PD-L1* expression. Once patients develop TKI resistance, ICI immunotherapy still remains an alternative option. A combination of TKI targeted therapy and ICI immunotherapy may raise safety concerns. For patients who lack TKI driver gene mutation who truly need and tolerate combined therapy well, immediate chemoimmunotherapy is recommended for optimal disease control and improvement; otherwise, a sequential regimen of single-agent immunotherapy followed by chemotherapy at progression is recommended. Nevertheless, appropriate sequencing paradigms between chemotherapy and immunotherapy still need investigation. To date, pembrolizumab with or without chemotherapy is the priority in the selection of ICI immunotherapy in NSCLC, whereas atezolizumab or durvalumab/chemotherapy is highly recommended in SCLC. Optimal management of the unique toxicities associated with ICI immunotherapy is also critical for the prognoses. Future direction of ICI therapy should focus on how to administer right immunotherapy regimens to the right immune microenvironment at the right time with minimum irAEs. It may encompass several aspects including searching for ideal biomarkers for therapeutic efficiency, determining the best criteria for response evaluation, solving primary and secondary resistance, preventing irAEs, and investigating optimal modes of ICI immunotherapy. In summary, ICI immunotherapy has established a novel treatment paradigm by remodeling the SOC for lung cancer. Despite this, tremendous endeavor is still warranted to enhance the outcome of patients with such intractable disease.

## Author Contributions

WX and other authors performed literature search, writing, and revising of the manuscript. All authors contributed to the article and approved the submitted version.

## Funding

The training program of leading talents of Pudong health system, Shanghai( Grant Number: PWRl2017-01); The training program of key department of Pudong health system, Shanghai( Grant Number: PWZzk2017-18).

## Conflict of Interest

The authors declare that the research was conducted in the absence of any commercial or financial relationships that could be construed as a potential conflict of interest.

## Publisher’s Note

All claims expressed in this article are solely those of the authors and do not necessarily represent those of their affiliated organizations, or those of the publisher, the editors and the reviewers. Any product that may be evaluated in this article, or claim that may be made by its manufacturer, is not guaranteed or endorsed by the publisher.
